# Supplementary material for: Are carbon emissions trading and green financial instruments synergistic? -Comprehensive quantitative research based on content analysis
Source: PLoS One. 2024 Mar 7;19(3):e0298601. doi: 10.1371/journal.pone.0298601 (PMC10919601; doi:10.1371/journal.pone.0298601)
Supplement: S1 Data — (ZIP) [file pone.0298601.s001.zip › Data/地级市Z_F工作报告.zip!/┴╔─■/╜⌡╓▌╩╨/└┤╫╘╣1⁄2╓┌║┼í╛┬φ┐╦╩2╛▌═°í┐_2005.docx]

各位代表：

　　现在，我代表市政府向大会报告工作，请予审议，并请市政协各位委员提出意见。

　　一、２００４年工作回顾

　　２００４年是实施振兴东北等老工业基地战略的起步之年，也是我市改革发展取得显著成绩的一年。在市委的领导下，市政府团结和依靠全市人民，深入贯彻党的十六大和十六届三中、四中全会精神，全面落实科学发展观，以建设辽西区域性中心城市为目标，抢抓机遇，开拓创新，大力实施“工业立市”、“以港兴市”和“开放牵动”三大战略，保持了国民经济的快速发展和各项社会事业的全面进步，基本完成了市人大十三届二次会议确定的各项任务。

　　２００４年，初步核算全市生产总值实现３３７亿元，按可比价格计算，增长１６．１％。其中，第一产业增加值８５亿元，增长１０．８％；第二产业增加值１３１亿元，增长２１．５％；第三产业增加值１２１亿元，增长１４．４％。第二产业增加值占全市生产总值比重达到３８．９％，提高２个百分点。地方财政一般预算收入完成１４．３亿元，按可比口径计算，增长１３％；社会消费品零售总额完成１２１．１亿元，增长１４．６％；居民消费价格总水平上升１．６％；城镇居民人均可支配收入达到７９８５元，增长１１．３％；农民人均纯收入达到３４００元，增长１２．６％；各项存款余额、贷款余额分别比年初增加４０．４亿元和３６．７亿元。

　　（一）工业经济快速增长，成为拉动全市经济发展的主导力量

　　规模以上工业总产值实现３４６．４亿元，增长３０．８％；实现利税２４亿元，增长４０．８％。更新改造投资力度加大。工业技术改造投资达到１４．７亿元，增长４２．７％。续建和新开工１０００万元以上项目６０个。攀钢锦州钛业钛白粉年生产能力达到１．５万吨，石油化工公司煤代油、元成生化公司６０万吨玉米、汉拿电机公司１４０万台汽车发电机起动机、锦恒公司２０万套安全气囊等一批项目竣工投产。已有１２个重大项目被列为国债支持项目。工业经济结构和布局进一步优化。石油化工、新型材料、农产品深加工三个优势产业和汽车零部件、电子信息两个新兴产业继续向规模化、集群化方向发展，“三二四”产业格局框架基本形成。规模以上工业企业高新技术产品产值完成７２．５亿元，增长３６％。临港工业保持快速增长，韩华电装等一批项目落户经济技术开发区。县（市）区工业增长较快，规模以上工业总产值增长４１．６％。一批骨干企业迅速成长。预计新增规模以上企业９０户，亿元以上企业１１户，５亿元以上企业１户。锦州石化公司实现工业总产值１５６亿元，增长３５．４％，占全市规模以上工业总产值比重达到４５％。国有企业改革继续深化。完成７户国有中小企业改制，改制面累计达到８７．２％。国有大中型企业主辅分离任务完成９７．５％。民营工业快速健康发展。实现增加值１０４亿元，增长３３．７％。新增规模以上民营工业企业１３１户。乡镇工业企业增加值实现９４．８亿元，增长３３．９％。

　　（二）农业和农村经济结构进一步优化，农业丰产农民增收

　　农业结构进一步优化。粮食总产量创历史最高水平，达到１９４万吨，增产６４万吨。农业总产值预计实现１７２亿元，增长１０．４％。林牧渔业产值占农业总产值比重达到５５％，提高１．６个百分点。新建各类农产品生产基地７０个，“六个百万”工程建设任务基本完成。新增绿色食品和无公害农产品认证２６个。新建农业科技园区１３个。农业产业化和工业化步伐加快。农村二、三产业新上５００万元以上项目１０５个。新增规模以上农业产业化龙头企业１７户。规模以上农产品加工企业实现营业收入３０亿元，增长２５％。转移农村剩余劳动力３万人。农村生产条件和生态环境进一步改善。新建水源工程１３００处，新增灌溉面积１０．３万亩。治理水土流失面积２１．４万亩。完成人工造林作业面积５５万亩，森林覆盖率达到２１．２％。实施人工增雨作业１９次。农业机械化水平达到５４％。农村各项改革不断深化。全面贯彻落实粮食直补、土地延包、税费改革等政策，农业税税率下调３个百分点，取消农业特产税，农民人均减负３７元。土地市场治理整顿取得阶段性成果，基本农田保护率达到８７．７％。完善了粮食储备制度，粮食购销总量在全省名列前茅。

　　（三）第三产业升级步伐加快，财政金融运行平稳

　　物流业蓬勃兴起。渤海、恒大和华联物流投入运营，恒大物流建立了辽西首家保税仓库。专业批发市场规模进一步扩大，宏发建材商城二期工程竣工投入使用。药品配送和监督网络延伸到县乡村。消费市场繁荣活跃。大型商业企业销售额创历史新高，新增连锁店铺３０家，新华广场、东方庭院综合市场开工建设。大厦宾馆完成改造并投入使用，金厦国际饭店等相继开业。建立了食品安全检测体系，实施了食品市场准入制度。旅游业健康发展。辽沈战役纪念馆改陈改造工程竣工开馆，大广济寺古建筑群维修示范工程开工建设。医巫闾山及北镇庙申遗工作继续推进。旅游总收入达到２２．８亿元，增长２６．７％。新兴第三产业取得新的突破。通信运营收入实现１０．８亿元，增长７．９％。城域光纤网已覆盖全市，互联网用户超过５．２万户。咨询、中介等行业发展进一步加快。

　　财政实现收支平衡。各级财税部门努力克服出口退税、下调农业税税率等政策性减收因素影响，强化税费征管，实现了财政、税收（基金）收入稳定增长。多方筹措资金，保证了公教人员国家标准工资及教育、科技、社会保障等法定支出和重点项目建设支出。深化部门预算编制、政府采购、国库集中支付、收支两条线等项改革，为构筑公共财政框架奠定了基础。金融保持平稳运行。金融资产质量进一步改善，不良贷款率下降４．３个百分点。农村信用社改革全面展开，市商业银行成功收购了１２家城市信用社并完成了机构整合。加大金融风险防范工作力度，保持了良好的金融秩序。

　　（四）基础设施建设力度加大，城市形象明显改观

　　城区改造力度进一步加大。编制完成《锦州市城市空间发展战略规划》并通过评审。投入资金１亿元，新建和大修城区道路１０５条，城区道路交通状况明显改善。完成了南站广场、垃圾处理厂主体、动物园一期等工程建设。城区房地产开发竣工面积５９万平方米，其中棚户区改造３．２万平方米。新增煤气用户１．１万户，解决了大凌里等２４７５户的供气遗留问题；新增供水、供暖面积７２万立方米和１４１万平方米；新增公交线路６条。完成南山绿化一期工程，植树造林１２００亩。城区植树造林６４万株，人均公共绿地面积上升到７．５平方米。重点项目建设进展顺利。锦州港建设步伐明显加快，２５万吨油品泊位开工建设，主航道疏浚工程进展顺利，２个７万吨级散杂货泊位竣工投入使用。港口吞吐量达到２４５５万吨，增长４３．８％，高于全国平均增幅２２个百分点。渤海大道开工建设。锦州中心渔港二期工程进展顺利。农村电网二期改造工程全部竣工。完成村村通油路工程６５９公里，新增通油路村１５６个。锦凌水库、高天地方铁路等重大项目前期工作取得实质性进展。市容环境卫生综合整治成效明显。马路市场、街路卫生、广告牌匾、占道经营及脏乱小区等得到进一步清理、整顿和规范。城市８０％以上天数空气质量达到国家Ⅱ级标准。

　　（五）对外开放工作取得新进展，外贸出口创历史最好水平

　　新批外商企业６４户；合同外资额３．１亿美元；实际利用域外资金２３．３亿元。招商引资重点更加突出。结合我市实际，把招商重点转向韩日港台等国家和地区，成功举办和参加了第六届锦州经贸洽谈会、锦州－香港投资说明会、锦州－韩国经贸活动周等境内外招商活动。对外开放质量明显提高。高新技术项目增多，外商投资进一步向商贸和基础设施领域延伸，日电铁合金、单晶硅切片等项目落户锦州。经济技术开发区对外开放窗口作用进一步增强，实际利用外资１８６０万美元。招商引资环境不断改善。第三次行政审批制度改革顺利推进，开展了窗口首席代表审批制，服务水平明显提高。外商投诉案件＂件件有着落、事事有回音＂。全面实施＂大通关＂建设，口岸环境进一步改善。出口创汇大幅度增长。实际出口创汇３．６亿美元，增长１４３．６％。出口额１００万美元以上的企业达到３６户。外派劳务人员２０４３人次，增长１３％。

　　（六）就业再就业工作取得新突破，社会保障体系进一步完善

　　全面落实就业再就业各项政策。积极开展就业援助，建立了就业实名登记制度，登记率达到９８％。新劳动力市场正式投入使用，在全省率先实现零散务工人员退路进厅。开发公益性岗位８５５５个，城镇劳动力实现就业和再就业７．２万人。进一步加强社会保障体系建设。养老、失业、医疗保险分别扩面１万人、２万人和４．７万人，养老保险社会化发放率和足额发放率均达到１００％。城镇居民最低生活保障人数达到６．８万人，城区保障标准每人每月增至１８０元，各县（市）也分别有所提高。高度重视困难群体救助工作。转移支付民政＂三项资金＂４７１９万元，为城区困难群众减免取暖费近８００万元，为农村贫困户、农村残疾人困难户分别建房１９０户和１８０户。全年筹集下拨救灾救济款（物）１０１２万元，救助困难群众２２万人次。农村贫困人口减少２万人。偿还拖欠农民工工资５８６９万元。

　　（七）各项社会事业协调发展，和谐锦州建设取得新成绩

　　大力推进科技进步。取得市以上科技成果４４项，科技进步对经济增长贡献率达到４８．８％。第八届中国（锦州）北方农业新品种新技术展销会规模进一步扩大，签订合作项目１１５个，协议金额１５．８亿元。驻锦大专院校、科研院所与地方经济建设联系更加紧密，科研成果转化率进一步提高。坚持人才强市，出台了《关于进一步加强人才工作的若干政策规定》，辽西人才市场正式挂牌。教育事业稳步发展。撤并中小学１４３所，新建九年一贯制学校１０所，中等职业技术学校招生增长２７．３％。城区中小学课程改革实验工作走在了全国前列。接收锦州铁路分局所属普通中小学１２所。公共卫生体系建设进一步加强。传染病医院、疾病预防控制中心、紧急救援中心３个国债项目基本完成。文化体制改革有力增强了文化事业的内在活力。组建了锦州日报报业集团和锦州广播电视总台。京剧《酒魂》等一批剧目分别在省和国家获奖。辽西文化古玩商城进入全国４２个文化产业示范基地行列。省十运会筹备工作顺利推进，完成了会徽、会歌、吉祥物的征集评选工作。＂双拥共建＂活动卓有成效。全面落实拥军优属各项政策，驻锦部队在城市建设、城乡绿化、精神文明建设等方面都做出了突出贡献。民族宗教工作取得新的进展。档案信息化建设得到加强。人口自然增长率控制在１．６２‰。

　　（八）民主法制建设进一步加强，保持了社会基本稳定

　　自觉接受人大法律监督、工作监督和政协民主监督，坚持政府与人大代表、政协委员联系制度。全年办复市人大议案１件，人大代表建议和政协提案５４４件，办复率和满意率分别达到１００％和９９．４％。全面贯彻落实《行政许可法》，对行政许可进行了全面清理。认真做好行政复议工作，结案率达到９２％。行政监察工作进一步加强，出台了《关于党政机关及其工作人员损害经济发展环境违纪行为的处分规定（试行）》等制度。坚持依法治市，＂四五＂普法工作取得新的成绩。积极开展严打整治斗争，破获了一批重特大案件，社会治安防控体系框架已经形成。妥善处理群众来信来访和市民投诉，及时预防和化解各种矛盾，保持了社会稳定。加强生产、交通和消防安全管理，安全生产形势总体平稳。

　　各位代表，２００４年各项工作取得的成绩，是省委、省政府亲切关怀的结果；是市委正确领导和人大、政协有效监督，各民主党派、工商联及各人民团体大力支持的结果；也是全市人民解放思想、励精图治、团结奋斗、共同努力的结果。在此，我代表市政府向全市广大工人、农民、知识分子和干部，向人民解放军驻锦部队指战员、武警官兵和公安干警，向中省直各部门、各单位，向所有关心、支持锦州发展的海内外朋友以及社会各界人士表示诚挚的感谢！

　　回顾一年的工作，我们必须清醒地认识到，在我市经济社会生活中仍然存在着许多困难和问题。主要表现在：固定资产投资不足，经济发展缺乏后劲，全社会固定资产投资完成７２．１亿元，比上年略有下降；开放度不高，招商引资水平较低，按新口径计算，实际利用外资４９２８万美元，与年初计划还有一定距离；财政增长缓慢，低于全省平均增长水平，收支矛盾突出，特别是县乡两级财政支付能力不强；软环境建设还有较大差距，政府职能转变还不到位，机关工作作风有待进一步转变。这些问题，我们将认真加以解决，以不辜负全市人民对政府的期望。

　　二、２００５年工作安排

　　２００５年是＂十五＂计划的最后一年，也是加快区域性中心城市建设、推动锦州老工业基地全面振兴的关键一年。当前我市已进入经济社会快速发展的重要机遇期，国家实施振兴东北等老工业基地战略，锦州已经得到国债项目等方面的有力支持，并有可能争取到更大的支持；省委、省政府对锦州高度重视和信任，积极支持锦州区域性中心城市建设；市委九届七次、八次全会确定的发展战略，完全符合锦州实际，已经在全市上下形成共识；全市广大干部群众求新、求变、求发展的愿望十分强烈，形成了推动经济社会快速发展的坚实基础和强大内在动力；锦州的固有优势更加明显，多年来积累的巨大能量，将在今后三年集中释放。这些如能充分把握，必将形成强大的发展合力。但是，机遇与挑战并存，机遇稍纵即逝，挑战咄咄逼人，不进则退，慢进亦退。我们必须立足当前，着眼长远，抢抓机遇，乘势而上，加速辽西区域性中心城市建设步伐。

　　２００５年，我市经济社会发展的总体要求是：以邓小平理论和＂三个代表＂重要思想为指导，认真贯彻中央、省经济工作会议和市委九届七次、八次全会精神，全面落实科学发展观，围绕建设辽西区域性中心城市的目标，深入实施＂工业立市＂、＂以港兴市＂和＂开放牵动＂三大战略，推进工业经济发展，优化农业和农村经济结构，提升第三产业现代化水平，大力发展社会事业，切实改善人民生活，为实现＂三年大变样、十年大振兴＂目标奠定坚实基础。主要预期目标是：全市生产总值增长１５％以上，其中一二三产业增加值分别增长９％、１８％和１６％。地方财政一般预算收入按可比口径增长１２％；全社会固定资产投资增长２５％；实际利用外资增长２０％，实际利用域外资金增长２０％；实际出口创汇增长１０％；社会消费品零售总额增长９％；物价总水平上涨幅度控制在４％以内；城镇居民人均可支配收入、农民人均纯收入分别增长９％和８％。

　　（一）坚持以项目为重点，推进工业经济跨越式发展

　　确保工业投资增长和重大项目实施。全市工业投资完成３０亿元，增长２０％。新开工投资１０００万元以上项目５０个，集中力量推进８英寸硅片等１２个国债项目和８万吨苯乙烯等１０个重点技术改造项目建设。努力争取新的国债项目。按照＂三二四＂格局调整工业结构。进一步做强做大石油化工、新型材料、农产品深加工三个优势产业和汽车零部件、电子信息两个新兴产业，加快传统产业升级。促进高新产业发展，高新技术产业产值达到９１亿元，增长２５％。大力发展民营工业。在资金、土地审批等方面予以倾斜，引导民营工业企业进入主导产业和重点工业区，推进企业向规模化、产业化、园区化和外向化方向发展。加快信用体系建设，搞好银企对接，切实解决民营企业融资难问题。民营工业实现增加值１３１．５亿元，增长２６％。做强做大一批骨干企业。积极实施名牌战略，提高市场占有率。鼓励和支持元成生化、沈宏实业、万得集团等企业扩大生产规模，新增１０亿元以上企业３户，５亿元以上企业４户，亿元以上企业４户，规模以上企业５０户。规模以上工业总产值达到４１６亿元，增长２０％。进一步优化工业布局。加快汤河子工业区振兴和临港工业区建设，规模以上工业总产值分别增长２０％和３２％，使之成为工业发展的两个增长极。做大县（市）区工业园区，加快形成产业集群。深化国有企业改革。重点抓好金纸、铁合金、重型等１０户企业改制，市属以下国有企业国有资本基本退出。

　　（二）重视解决＂三农＂问题，确保农业和农村发展的良好势头

　　大力推进产业化经营。以打造绿色品牌为标志，以各具特色的沿山、沿路、沿海经济板块为载体，实施品牌战略，实行标准化生产，做强做大粮食、蔬菜、水果、水产、畜牧、油料加工和专业批发市场等８０个农业产业化龙头企业，壮大提升农产品生产加工体系，健全完善农产品流通体系。农村二三产业增加值增长２３％。进一步优化农业结构。全面提升＂六个百万＂工程质量和效益，积极推广龙头企业带基地连农户的生产经营模式，突出抓好＂一牧两水一菜＂四大产业１００个市级以上标准的农业科技示范园区和绿色、无公害生产基地建设。不断改善农村生产生活条件和生态环境。启动大小凌河地下水资源保护工程，防止海水倒灌。全面推进开发云水资源、植树造林等１０项效益工程建设，实施水源工程１０００处，新增灌溉面积１０万亩，治理水土流失面积１７万亩。实施绿化荒山、绿化河滩、绿化通道、绿化村屯等＂四绿工程＂。开工建设南凌一级渔港，锦州中心渔港投入使用。贯彻实施《农业机械化促进法》，推进农机化发展步伐。加快农村剩余劳动力转移。重点抓好政策落实、用工订单、技能培训和跟踪服务，加快农村剩余劳动力向非农产业和城镇转移，全年转移劳动力５万人。深化农村体制改革。按照省统一部署全部取消农业税，进一步减轻农民负担。继续深入开展土地市场治理整顿，加强基本农田保护，节地挖潜，确保重点建设项目用地需求。继续深化粮食供销流通体制改革。

　　（三）提升现代服务业水平，巩固辽西商贸中心地位

　　全面提升商贸流通业现代化水平。加快宏发建材商城三期工程、新华广场等项目建设。重点构建凌南新区购物体系。积极引进沃尔玛等国内外著名大型和特大型商业企业，发展连锁店铺１８家。鼓励大商、中百、华联等大型零售企业资本扩张，不断开拓城乡市场。建立食品卫生保障体系。加强市场监管，严格执行食品市场准入制度。积极推进＂三绿＂工程，提倡绿色消费，培育绿色市场，开通绿色通道，保证市民的食品安全。积极发展现代服务业。大力发展物流配送、电子商务、通信网络、中介信息等新型现代服务业，完成渤海物流二期、华联物流二期工程和六陆公司２个１０万吨石油储备罐建设，积极推进恒大物流申请建设保税中心工作。大力发展旅游产业。全方位开拓旅游市场，重点发展＂红色之旅＂游、滨海度假游、山海风光游。旅游总收入实现２５．５亿元，增长１１．８％。促进金融产业健康发展。深化农村信用社和城市国有商业银行改革工作。完善商业银行经营机制，提高资产质量。积极引进全国性股份制商业银行来锦设立分支机构。各金融部门要进一步加大对地方经济建设的支持力度。

　　（四）加大招商引资力度，实现对外开放新突破

　　提高招商引资项目成功率。重点推进高果糖、真空开关管等１０个重点招商引资项目落户。每个县（市）外资实际到位额要达到４００万美元以上，城区７００万美元以上，凌南新区９００万美元以上，经济技术开发区２８００万美元以上。加快境内外招商活动频率。以韩国、日本、香港、台湾等国家和地区以及长三角、珠三角等国内较发达地区为重点，小队伍、多批次，全方位、高质量地开展境内外招商活动。举办好在韩国、香港等地的招商说明会和第七届锦州经贸洽谈会。扩大招商引资领域。鼓励外资向高新技术产业、农业、服务业、城市公用设施和教育、文化、卫生等领域投入，引进现代体制增强内在活力，引进先进理念提升管理档次，引进资金促进经济与社会协调发展。抓好项目库建设。按照国际惯例精心包装储备一批符合产业转移趋势、具有较强牵动作用的大项目和一批对接性强、操作灵活的中小项目，切实增强项目的吸引力和针对性。确保市本级＂精品＂包装项目不少于３０个，每个县（市）区不少于１０个。充分发挥企业招商引资主力军作用。引导鼓励各种所有制企业主动与国外大公司合资合作。支持锦泰、圣戈班、平安印染等现有＂三资＂企业增资扩股，力争培育１０户产值超亿元的骨干企业。优化出口产品结构。以扩大高新技术产品和机电产品出口为重点，努力提高出口产品附加值。实际出口创汇４亿美元。外派劳务２３００人。

　　（五）强化财源建设，优化财政支出结构

　　巩固壮大现有财源，积极培植后续财源。采取改革挖潜、招商引资等办法，盘活资产存量，狠抓项目建设，进一步优化工业主体财源，发展农业基础财源，扩大商贸财源，培育城市经营财源，壮大富民强县的税收产业群。每个县（市）区都要着重扶持１－２个支柱产业，新增纳税超百万元企业５户；每个乡镇都要新上１－２个税源型企业。用足、用好各项扶持政策，积极争取资金支持。充分利用国家和省对县调整财政体制的相关政策，采取融资担保、贴息、核呆、以奖代补等方式，吸引社会资金投入优势产业、高新技术产业、税源型企业。大力实施重点乡镇帮扶计划，力争使３－５个乡镇进入全省百强乡镇。强化税收征管。巩固税收责任制，完善税收征管系统，营造公平诚信的纳税环境。严厉打击偷逃骗税行为，禁止乱拉税源、乱拉管户，清理核查福利企业、校办企业，最大限度地减少跑冒滴漏，确保财政收入的完整性。强化非税收入管理，扩大财政收入规模。严格控制支出。树立过紧日子思想，严禁铺张浪费。调整和优化财政支出结构，继续扩大国库集中支付和政府采购范围。坚决压缩一般性支出，合理调减专项支出，重点保证工资发放、社会保障和救灾救济等项支出，确保政权运转和社会稳定。

　　（六）加强基础设施建设，不断提升城市品位

　　继续改善老城区的人居环境。进一步实施城市街路改造工程，新建大修主次干道１５条、小街小巷１００条，新安装７条以上街路路灯。完成青年公园改造和吉祥里等３片以上棚户区改造建设任务。推进水源、气源、热源建设及管网改造，新增煤气用户１万户、集中供热面积１００万平方米。新增公交线路２条。加速城市南扩重点工程的起步工作。认真做好城市总体规划和土地利用规划局部调整的编制、报批工作。加快渤海大道工程建设，完成路基、隧道和大中桥及互通立交桥工程。完成市府广场建设任务。完善凌南新区主干路网建设。新建女儿河南岸截流干管１２公里，启动小扒沟改造工程。以凌南新区为重点，全面激活房地产市场，开发建筑面积５０万平方米。加快南山绿化步伐，全民动员、全城参与，春、雨、秋三季再掀植树高潮。实施新建街路绿化工程。加大能源交通等重点基础设施建设力度。锦州港建设投资１０亿元以上，确保２５万吨级油品泊位、主航道拓宽工程和２个年吞吐量４０万标箱的集装箱泊位投入使用。开工建设２个１０万吨级散杂货泊位和１个３万吨级油品化工泊位。港口吞吐量力争达到３０００万吨。加快开辟锦州港第二战场，完成锦州港二期工程开发方案设计工作。全面启动高天地方铁路增能改扩建工程，力争年内通过能力达到１０００万吨。完成１０２国道大凌河至沟帮子段等道路大修工程，继续实施村村通油路工程。建成污水处理厂并完成整体经营权转让，垃圾处理厂投入使用。广泛开展争创国家卫生城活动。继续开展环境卫生特别是住宅小区综合整治，巩固和完善＂门前三包＂责任制，加快城管和环卫体制改革，建立城市管理的长效机制。积极推进环保模范城争创工作。

　　（七）扩大就业和再就业，完善社会保障体系建设

　　全力做好就业再就业工作。坚持就业工作＂四个优先＂的原则，建立就业再就业责任分担体系，全面落实再就业各项优惠政策，搞好劳动力资源普查，建立实名制工作长效机制。强化职业技能培训和创业培训，建立市、区、街、社区四级就业信息网络系统。进一步打造品牌劳动力市场。安排就业再就业５．４万人，其中＂４０５０＂人员实现就业５２００人；登记失业率控制在５．７％以内。加强劳动力监察和劳动关系协调，保障劳动者合法权益。健全社会保障体系。进一步完善养老、失业、医疗保险，积极推进工伤和生育保险。加大基金征缴力度，确保离退休人员基本养老保险金按时足额发放。依法扩大各项社会保险覆盖面，养老、失业、医疗保险参保人数分别达到２８万人、３９万人和３８．５万人。积极推进住房公积金制度建设。建立健全城乡社会救助体系。继续完善城市低保制度，建立和推进农村低保制度。积极开展分类救助，增强救助的针对性和有效性。制定出台《锦州市城市低保对象重大疾病医疗救助保险办法》，建立城乡贫困群众大病救助制度。实施突发应急救助、日常生活、住房、就学及冬季取暖援助等政策措施，妥善解决涉及弱势群体的热点难点问题。努力做好残疾人康复、就业、维权等各项工作。加强监管，确保民政＂三项资金＂足额发放到位。加快养老产业发展。加大扶贫工作力度。建立集中捐赠与经常性扶贫相结合、输血式扶贫与造血式扶贫相结合的长效机制，突出抓好３０个贫困村＂整村推进＂、３００户移民搬迁和定点帮扶工作。充分发挥＂扶贫超＂市作用，发动社会力量，使扶贫帮困活动经常化。

　　（八）发展各项社会事业，努力构筑和谐社会

　　提高科技创新能力。深化科技体制改革，加大重点领域、重点产业和重点项目科技投入，科技对经济增长贡献率达到５１％。举办好第九届中国（锦州）北方农业新品种新技术展销会和第六届海外学子创业周等活动。切实加强人才工作。紧紧围绕老工业基地振兴开展人才服务工作。大力推进事业单位人事制度改革，加速事业单位的社会化进程。积极发展教育事业。进一步调整教育布局，撤并农村中小学校７１所，改造危房面积５．７万平方米以上，城区中等职业学校整合为１２所。黑山县、义县完成巩固＂普九＂任务，北宁市、凌海市和太和区完成提高＂普九＂任务，城区学校２０％达到＂双高＂标准。农村初中辍学率控制在３％以下，普通高中扩大招生２０％。按照一流校舍、一流师资、一流教学水平的标准，在凌南新区建成一所高标准九年一贯制义务教育学校。开工建设辽宁工学院滨海校区一期工程。大力发展卫生事业。理顺乡镇卫生院管理体制，重点加强１８所乡镇中心卫生院和６所偏远地区卫生院建设。积极抓好大病统筹合作医疗试点，推进乡镇防保体制改革，逐步建立农村医疗救助制度。进一步完善疾病预防控制体系、医疗救治体系和卫生监督执法体系建设。完成传染病医院二期工程。加快市中医院向综合性医院发展步伐。不断繁荣文化事业。进一步深化文化体制改革，大力发展文化产业。加强优秀剧目的市场化经营演出。搞好特色群众文化活动，策划创办冬季庙会、夏季笔架山海会，春季农展会增加文化内容，继续办好秋季第七届民间文化节。积极推进医巫闾山及北镇庙、义县奉国寺申遗工作。完成市图书馆收尾工程并争取早日开馆。努力做好广播电视、体育、人口和计划生育等项工作。搞好有线电视网络资源整合，加快广播电视由模拟向数字整体转换进程。坚持市场化运作与政府推进相结合，精心做好省十运会各项筹备工作，全面提高竞技体育和群众体育水平。加强人口和计划生育综合改革与服务，搞好１％出生人口普查，人口自然增长率控制在３．１９‰以下。认真做好民族、宗教、侨务、档案等项工作。不断加强＂双拥＂工作。全面落实各项政策，创造性地开展＂双拥＂工作，密切军政军民关系，巩固＂双拥模范城＂成果。进一步加强社区建设。改革社区干部队伍管理体制，强化社区调解服务功能。多渠道筹集资金，努力完成社区＂一站四室＂基础设施建设。

　　努力维护社会稳定。大力推进社会治安综合治理，加强社会治安防控体系建设，加大突出治安问题整治力度。坚决打击惩治邪教组织，扫除＂黄、赌、毒＂等社会丑恶现象。继续加大＂网吧＂整治力度，净化校园周边环境。巩固深化警务体制、勤务方式改革，建立健全预警和快速反应机制，保持打击刑事犯罪的高压态势，加大对恶性案件的侦破力度。进一步完善应急突发事件预案，积极稳妥有效地处置突发事件和群体性事件。认真搞好普法宣传，完成＂四五＂普法总结验收工作。做好法律援助工作，维护人民的合法权益。坚持信访工作党政＂一把手＂责任制、信访责任追究制、主要领导包案和信访公开接待日制度，切实做好群众来信来访工作，努力化解各类社会矛盾。健全理顺安全生产监管体制，落实安全生产责任制和责任追究制，抓好交通安全、矿山安全、消防安全等专项整治，坚决遏制重特大事故的发生。

　　（九）加强政府行政能力建设，创造良好发展环境

　　完成今年各项任务，各级政府必须按照中央和省、市委的统一要求，深入开展保持共产党员先进性教育活动，切实加强自身建设，不断提高服务水平。

　　不讲空话，建设依法诚信政府。坚持实事求是、脚踏实地的原则，不讲空话、大话和套话。依法行政并自觉接受人大法律监督、工作监督和政协民主监督，认真办理人大议案、代表建议和政协提案。深入贯彻《全面推进依法行政实施纲要》和《行政许可法》，严格按照法定权限和程序行使权力、履行职责。理顺行政执法体制，积极稳妥做好相对集中处罚权工作。进一步做好行政复议工作。建立坚强有力的行政指挥系统，反应迅速，令行禁止，确保各项工作＂定一项落一项，项项落实；干一件成一件，件件成功＂。

　　不图虚名，建设勤政务实政府。各级政府工作人员特别是领导干部要牢固树立正确政绩观，真正扑下身子、放下架子，深入基层、深入群众，掌握真实情况，解决实际问题，力求把涉及全局和人民群众利益的每一项工作做好。切实转变作风，提高服务意识，加大服务力度，同时要主动向上级争取政策、项目和资金，为地区经济发展争取更多的支持。进一步修订政绩考核办法，加大对重点工作落实情况的考核力度。建立新闻发布会制度，适时向群众发布各类信息。努力把政府经济管理职能转到服务和改善环境上来，创造统一开放、公平透明、竞争有序的市场运行体系。

　　不谋私利，建设廉洁高效政府。各级政府工作人员要廉洁自律，严格遵守反腐倡廉的各项规定，自觉接受党和人民群众的监督，经受住权力和金钱的考验。严格落实《行政执法责任制》，切实加强公共财政审计、绩效审计和党政领导干部任期经济责任审计。全面开展＂机关作风建设年＂活动，切实提高机关行政效能，对损害经济发展环境的违纪行为坚决严肃处理。继续深化第三次行政审批制度改革，实行集中受理、并联审批、一窗式服务，确保行政审批事项压缩到３００项以内。大力推行电子政务，切实提高行政效率。

　　今年是＂十五＂计划的最后一年，也是编制＂十一五＂规划的重要一年。我们要深入贯彻市委九届八次全会精神，以科学发展观为指导，集中民智，反映民意，科学确定发展目标、发展步骤和具体措施，高标准、高质量地完成好＂十一五＂规划编制工作。同时，要认真搞好第一次经济普查工作。

　　各位代表，锦州已经进入加快发展的关键时期，全市人民多年企盼并为之不懈奋斗的经济振兴、社会繁荣的美好画卷已经展现在我们面前。任务艰巨，责任重大。我们有信心不辱使命，在市委的坚强领导下，依靠和带领全市人民抢抓机遇，积极进取，求真务实，开拓创新，为全面建设辽西区域性中心城市做出新的贡献！
